# DNA–Gold Nanoparticle Dumbbells: Synthesis and Nanoscale Characterization

**DOI:** 10.3390/nano15201583

**Published:** 2025-10-17

**Authors:** Esraa Hijaze, Liat Katrivas, Zakhar Reveguk, Shachar Richter, Alexander B. Kotlyar

**Affiliations:** 1 Department of Biochemistry and Molecular Biology, George S. Wise Faculty of Life Sciences, The Center for Nanoscience and Nanotechnology, Tel Aviv University, Ramat Aviv, Tel-Aviv 69978, Israel; esraahijaze@mail.tau.ac.il (E.H.); liatkatrivas@mail.tau.ac.il (L.K.); zakhar@mail.tau.ac.il (Z.R.); 2 Department of Materials Science and Engineeering, The Iby and Aladar Fleischman Faculty of Engineering, The Center for Nanoscience and Nanotechnology, Tel Aviv University, Ramat Aviv, Tel-Aviv 69978, Israel; srichter@tauex.tau.ac.il

**Keywords:** DNA, nanoparticles, DNA-nanoparticle dumbbells, AFM, TEM, DNA-nanotechnology

## Abstract

We report an efficient, high-yield method for synthesizing dumbbell-shaped conjugates composed of gold nanoparticles (AuNPs) connected by double-stranded (ds) DNA. The dsDNA, bearing terminal thiol groups, was covalently attached to two AuNPs to form uniform constructs comprising either 15 nm or 25 nm particles bridged by 38 base pairs (bp) or 100 bp dsDNA. The dumbbells were purified by gel electrophoresis and exhibited high stability, remaining intact for several days in pure water or buffers at ambient temperature. Deposition onto solid substrates followed by drying, however, led to their partial structural collapse. TEM imaging showed that deposition on carbon grids typically yielded dumbbell structures with interparticle gaps of only 1–2 nm, suggesting that the dsDNA bridge contracts during deposition and drying. However, deposition on polylysine-coated mica for AFM imaging preserved the native geometry, with the gaps consistent with the expected DNA length. Our results reveal that deposition significantly affects the structure and integrity of dsDNA bridges in dumbbell constructs, highlighting the importance of appropriate substrate and surface coating selection for reliable characterization of DNA properties in dried dumbbells.

## 1. Introduction

Gold nanoparticles (AuNPs) have become widely used components in nanotechnology, biosensing, and materials science due to their unique optical, electronic, and self-assembling properties (for reviews, see [1,2,3]). The particles’ surface can be readily functionalized with biomolecules, enabling the construction of highly programmable nanoscale architectures. Among the most versatile molecular tools for directing the spatial arrangement of AuNPs is DNA (for reviews, see [4,5]). In particular, short (tens of bases) double-stranded DNA (dsDNA) is capable of bridging nanoparticles with nanometer-scale precision. One particularly intriguing class of such nanostructures is AuNP dimers (or “dumbbells”), consisting of two gold nanoparticles connected by a single dsDNA molecule. These constructs have attracted considerable interest in a variety of fields, including DNA detection [6], plasmonics [7,8,9,10], nanoscale distance measurements [7,8], and molecular electronics [11]. Placing such dumbbells between electrodes allows direct measurement of the electrical conductivity of DNA. Reliable metal–metal contact between the nanoparticle surface and the electrodes ensures efficient electron injection into the DNA, thereby allowing a reliable measurement of its intrinsic conductive properties [11]. Electrical measurements on such AuNP-DNA dumbbells enabled investigation of the mechanisms of charge transport in DNA, providing evidence that the sugar-phosphate backbone may play a more crucial role in electron transfer than previously thought, challenging earlier models that emphasized the role of π–π nucleobase stacking [11]. These advances pave the way for ultrasensitive detection technologies, in which DNA–AuNP dumbbells act as functional elements for detecting trace amounts of DNA via electrical signals or for real-time monitoring of enzyme activity at a single-molecule level [12]. This approach holds promise for high-resolution, label-free DNA sequencing. In this context, conductive DNA–AuNP dumbbells may offer a more stable and biologically compatible alternative to the organic conductive polymers previously used for real-time electrical monitoring of DNA polymerase activity. Numerous studies have explored the formation and properties of AuNP-DNA dumbbells [6,7,8,9,10,11,13,14,15,16,17,18]. A common strategy for their preparation relies on conjugating single-stranded DNA (ssDNA) with terminal thiol groups to AuNPs, followed by gel electrophoresis-based separation of conjugates carrying different numbers of strands per particle. The conjugates are then annealed with a complementary ssDNA strand conjugated to another AuNP (which was also pre-purified by gel), yielding a dsDNA-bridged AuNP dimer. While effective for small nanoparticles (<10 nm) and long DNA strands (>100 bases), gel electrophoretic separation of the conjugates becomes increasingly inefficient when applied to larger particles or shorter strands.

To overcome this limitation, a variation of the strategy was developed, which involves hybridization of the short ssDNA strand with longer complementary strands before the electrophoretic separation. These extended conjugates can then be purified and later de-hybridized to yield the purified various AuNP-ssDNA conjugates [14,15]. Hybridization of two complementary conjugates produces dumbbell structures. However, this multistep procedure is relatively complex, typically gives low yields, and utilizes long oligonucleotides.

A potentially more straightforward alternative strategy involves the initial hybridization of two complementary ssDNA oligonucleotides, each bearing a terminal thiol group, to form a dsDNA with SH-groups at both ends. This dsDNA is then incubated with an excess of AuNPs, resulting in the formation of dumbbell structures with a dsDNA bridge. While this approach has been mentioned in the literature, for example, in the context of conjugating 10 nm AuNPs with a 16-bp SH-functionalized oligonucleotide duplex [16], no convincing experimental results were provided. Thus, while the strategy is conceptually promising, it has not been adequately validated or optimized for routine use, especially when working with large AuNPs and/or short DNA. Given the technological importance of such dumbbells, both for fundamental studies on DNA conductivity and for the development of advanced biosensing and single-molecule enzymatic monitoring systems, we undertook a detailed investigation of their formation, electrophoretic behavior, and properties.

Here, we present a simple, efficient, and high-yield procedure for the synthesis of AuNP–DNA dumbbells using either 15 nm or 25 nm AuNPs and dsDNA linkers of 38 or 100 base pairs (bp). The resulting constructs were purified by gel electrophoresis and analyzed by AFM and TEM to confirm their morphology. We further investigated the plasmonic properties of these constructs. We observed weak plasmonic coupling in the dumbbell in the case of 25 nm AuNPs linked by 38-bp DNA, whereas no coupling was detected for 15 nm AuNPs or the 100-bp linker. We also found that plasmonic coupling in the dumbbell structures can be influenced by protein binding, such as Cas12a, which interacts with the DNA bridge raising the possibility that enzyme-induced changes in dumbbell properties may provide a way to monitor alterations in DNA structure caused by DNA-dependent enzymes.

## 2. Materials and Methods

### 2.1. Materials

Unless otherwise stated, the reagents were obtained from Sigma-Aldrich (St. Louis, MO, USA) and were used without further purification. Oligonucleotides were purchased from Integrated DNA Technologies, Inc. (Integrated DNA Technologies, B.V., Leuven, Belgium) unless stated otherwise.

### 2.2. Synthesis of AuNPs

#### 2.2.1. 15 nm AuNPs

Spherical gold nanoparticles (~15 nm in diameter) were synthesized by citrate reduction of HAuCl_4_, following a modified Turkevich method [19]. Briefly, 200 mL of deionized water was added to a 500 mL Erlenmeyer flask under vigorous stirring, and a small volume of concentrated HAuCl_4_ solution (1–2 M) was introduced to achieve a final gold ion concentration of ~0.22 mM. The mixture was stirred at room temperature for 10–15 min, and transferred to a glycerol bath preheated to 140 °C. Once the solution reached boiling (~2 min), 3 mL of 34 mM trisodium citrate (filtered through an Amicon^®^Ultra Centrifugal Filter, 3 kDa MWCO) was quickly added under vigorous stirring. The reaction was considered complete when the color of the suspension no longer changed, typically after 5–7 min. The flask was then removed from the bath and allowed to cool to room temperature. The resulting AuNP suspension remained stable for several months under ambient storage conditions.

#### 2.2.2. 25 nm AuNPs

Gold nanoparticles were synthesized using a seed-mediated growth method, in which 15 nm AuNPs were enlarged in the presence of citrate and gold ions. The procedure was a slight modification of a previously published protocol [20]. Briefly, 200 mL of deionized water was added to a 500 mL Erlenmeyer flask, and a small volume of concentrated HAuCl_4_ solution (1–2 M) was introduced to achieve a final gold ion concentration of ~0.22 mM. The mixture was stirred at room temperature for 10–15 min. The solution was kept at room temperature for 10–15 min at ambient temperature. The seed solution was prepared separately by adding 40 μL of 1 M Na_3_-citrate (filtered through a 3 kDa Amicon^®^ Ultra centrifugal filter) dropwise to 50 mL of freshly prepared 15 nm AuNP seeds (optical density at 520 nm ~0.8) under vigorous stirring. The gold ion solution was then transferred to a glycerol bath preheated to 140 °C. Once the solution reached boiling (~2 min), the seed solution was rapidly added under continuous stirring. The reaction was considered complete when the suspension developed a stable wine-red color, typically after 5–7 min. The flask was then removed from the heat and allowed to cool gradually to room temperature. The resulting AuNP suspension remained stable for several months when stored under ambient conditions. The particle sizes, determined by AFM, were 15 ± 2 nm and 25 ± 3 nm. Particle concentrations were estimated spectrophotometrically using extinction coefficients of 2.7 × 10^8^ M^−1^ cm^−1^ at 520 nm for 15 nm AuNPs and 7.5 × 10^8^ M^−1^ cm^−1^ at 525 nm for 25 nm AuNPs [21,22,23].

### 2.3. Preparation of DNA Samples

The following DNA sequences were used in this study (5′→3′):

“100-1”—5ThioMC6-D/TT TCA GAC AAG ATT CAT CTA GCA CTG GCT GGA ATG AGA CTA TTG TTG AGA ACC TCC TGG CTA ATG TCT ATC ATC AGA TAA ACC ATC TGA AGA CAG TCC TG

“100-2”—5ThioMC6-D/CA GGA CTG TCT TCA GAT GGT TTA TCT GAT GAT AGA CAT TAG CCA GGA GGT TCT CAA CAA TAG TCT CAT TCC AGC CAG TGC TAG ATG AAT CTT GTC TGA AA

“38-1”—5ThioMC6-D/ACATAACTTACGTTAACAACCTCTGTTGCTTGGTTTTT

“38-2”—5ThioMC6-D/AAAAACCAAGCAACAGAGGTTGTTAACGTAAGTTATGT

Each oligonucleotide was dissolved in 300 µL of deionized water (DDW), vigorously mixed, and incubated at ambient temperature for 30 min. The solutions were then centrifuged at 14,000 rpm for 5 min using a benchtop Eppendorf centrifuge. The resulting supernatants were collected, and the concentrations of the oligonucleotides were determined spectrophotometrically using the following extinction coefficients (in M^−1^cm^−1^): 973,400 for “100-1”, 976,200 for “100-2”, and 353,400 and 395,200 for “38-1” and “38-2”, respectively.

Complementary 100-base or 38-base oligonucleotides were hybridized by mixing each strand pair at equimolar concentrations in 50 mM HEPES-K buffer (pH 7.5). The mixtures were heated to 90 °C in a dry bath incubator and left at this temperature for 10 min. Afterward, the heater was turned off and the tubes were allowed to cool gradually to room temperature within the device. The disulfide groups at the ends of hybridized 38 bp and 100 bp dsDNA in the hybridized were reduced during incubation with dithiothreitol (DTT). To do that, each of the above dsDNA types was incubated in 50 mM K-Pi buffer (pH 7.5) with 20 mM DTT for about 16 h at ambient temperature. Typically, the volume of the incubation is 100 μL, and the OD of the DNA at 260 nm is 200–400 AU. The incubation was then chromatographed on a NAP-10 column equilibrated with 20 mM citrate-K (pH 6.0) and 30 mM KCl. A fraction eluted in the void volume of the column (between 1.1 and 1.5 mL) was collected. Concentration of the dsDNA was estimated using extinction coefficients (in M^−1^cm^−1^) of 1,650,000 for 100 bp and 570,000 for 38 bp DNA, respectively. The eluted dsDNA can be stored at −80 °C for months.

### 2.4. Formation of Closely Spaced 25 nm AuNP Dumbbells

Dimers composed of 25 nm AuNPs were prepared as described in [24]: 10 mL of 25 nm AuNP suspension was placed in a 20 mL scintillation vial, and 40 µL of 1 M Tris-HCl (pH 8.0) was added in one aliquot under rapid stirring. After 5 min at ambient temperature, 100 µL of 1 M Tris-HCl (pH 8.0) was added in one aliquot under rapid stirring. Following 8 min of incubation, 50 µL of 0.1 M 5 mM Bis (p-sulfonatophenyl) phenylphosphine (BSPP) was added, and the mixture was stirred for an additional 5 min. The mixture was centrifuged in a 15 mL Corning tube at 4000 rpm for 1 h at 20 °C using a 5810-r Eppendorf centrifuge. The resulting pellets were resuspended in 60 µL of DDW. Ten microliters of this suspension were mixed with 0.2 µL of 0.1 M BSPP and glycerol, loaded onto a 2% agarose gel, and electrophoresed under standard conditions. The dimer band was excised and electroeluted into TAE buffer and concentrated in a 1.5-mL Eppendorf tube on a benchtop Eppendorf centrifuge for 5 min, 5000 rpm. The pellet was resuspended in 50 µL DDW. This purified dimer fraction was characterized by UV–vis spectroscopy.

### 2.5. Preparation of CRISPR-Cas12a

Alt-R™ L.b. Cas12a (Cpf1) Ultra and gRNA (Alt-R™ L.b. Cas12a crRNA) were purchased from Integrated DNA Technologies (IDT, Coralville, IA, USA). The sequences (5′→3′) of the guide RNA, crRNA, and target DNA were as follows: rUrA rArUrU rUrCrU rArCrU rArArG rUrGrU rArGrA rUrCrG rUrCrG rCrCrG rUrCrC rArGrC rUrCrG rArCrCrU;

TTTACGTCGCCGTCCAGCTCGACCT (sense DNA strand),

AGGTCGAGCTGGACGGCGACGTAAA (antisense DNA strand).

Conjugation of Cas12a with crRNA was conducted according to standard protocol: crRNA was dissolved in 10 mM Tris-HCl (pH 7.5) containing 0.1 mM EDTA-K at a concentration of ~100 μM and mixed with Cas12a (final concentrations: 10 μM Cas12a and 12 μM crRNA). The mixture was incubated at 25 °C for 30 min.

dsDNA target was mixed and hybridized as follows: The 25-nt sense strand and its complementary antisense strand (see above) were mixed at equimolar concentrations (~20 μM each) in 20 mM Tris-HCl (pH 7.5). The mixture was heated to 90 °C in a dry bath, which was then switched off, allowing the tube to cool slowly to ambient temperature over 4–5 h.

Conjugation of Cas12a–crRNA with dsDNA target (activation of Cas12a) was done by mixing the Cas12a–crRNA complex with the dsDNA target in buffer containing 20 mM HEPES-K (pH 7.5), 20 mM MgCl_2_, 150 mM KCl, 1% glycerol, 0.5 mM DTT, and 0.1 mg/mL BSA. Final concentrations of both Cas12a–crRNA and dsDNA were 1.5 μM. The mixture was incubated at 37 °C for 1 h.

### 2.6. Electrophoresis

Prior to electrophoresis, 10 µL of 87% glycerol was added to 120 µL of the incubation mixture (see above). The resulting sample was loaded onto a 2% agarose gel (~15 µL per lane) and run at 100 V for 1 h in an ice bath. TAE buffer was used both for gel preparation and as the running buffer.

### 2.7. Atomic Force Microscopy (AFM)

DNA–AuNP dumbbells were deposited onto polylysine-coated mica (PL-mica). The PL-mica was prepared by applying ~100 µL of 0.01% polylysine solution in DDW onto freshly cleaved mica and incubating for 10 min. The surface was then rinsed with DDW and dried under a gentle nitrogen (N_2_) flow. The dumbbell solution was diluted in DDW to a concentration corresponding to an optical density of 2–3 at 520 nm. A 10 µL aliquot of this solution was deposited onto the PL-mica, incubated for 1 min, rinsed with DDW, and dried under N_2_. AFM imaging was performed using a Solver PRO system (NT-MDT, Zelenograd, Russia) in semi-contact mode with 130 µm-long Si-gold-coated cantilevers (ScanSens, Munich, Germany), having a resonance frequency of 49–69 kHz. Acquired images were “flattened” by fitting each scan line to a second-order polynomial and subtracting the fit using Nova image processing software (version 3.5, NT-MDT, Moscow, Russia). Further image analysis was carried out using Gwyddion software (https://gwyddion.net/, accessed on 30 August 2025).

### 2.8. Transmission Electron Microscopy (TEM)

2 μL of DNA–AuNP dumbbells (2–3 OD at 520 nm) were deposited onto carbon-coated TEM grids (LC200-CU-CC; Electron Microscopy Sciences, Hatfield, PA, USA) pretreated with a 25% O_2_/75% Ar plasma for 7 s. After 2 min of incubation, the excess solution was removed from the grids using filter paper. The grids were further dried under vacuum for 15 min and plasma-treated again under the same conditions. TEM imaging was conducted using a TALOS microscope (ThermoFisher Scientific, Waltham, MA, USA) operating in bright-field mode at 200 kV.

### 2.9. Absorption Spectroscopy

UV-Vis absorption spectra were recorded under ambient conditions using a Scinco S-3100 spectrophotometer (Seoul, Republic of Korea).

## 3. Results and Discussion

DNA–AuNP dumbbells were prepared by mixing highly concentrated AuNPs with double-stranded DNA (dsDNA) containing terminal thiol (–SH) groups, previously treated with DTT, as described in Section 2.

**Figure 1:**
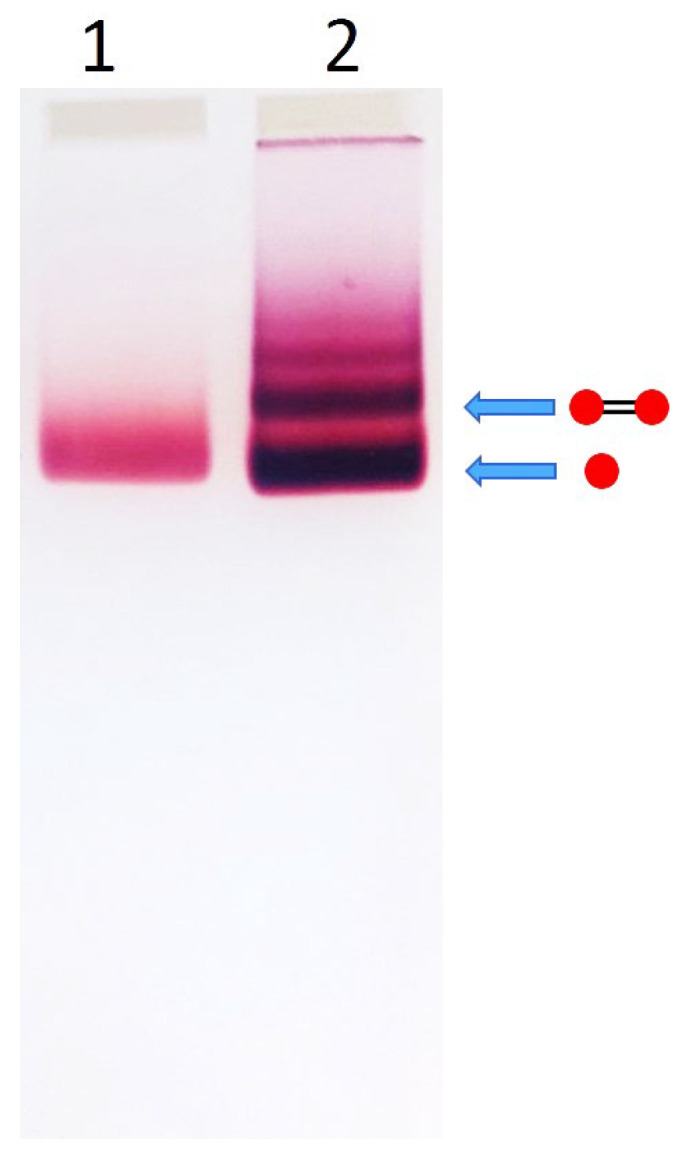
Electrophoretic separation of AuNP–100 bp DNA conjugates. Lane 1—0.45 μM 15 nm AuNPs. Lane 2—0.45 μM 15 nm AuNPs incubated with 0.45 μM 100 bp DNA for 16 h at RT in 5 mM HEPES–K (pH 7.5), 15 mM KCl. Electrophoresis was performed in 2% agarose gel (7 × 7 cm^2^) in TAE buffer at 100 V for 1 h in an ice bath, as described in Section 2. The drawings to the right of the gel illustrate the structures corresponding to specific bands (indicated by arrows). Red circles represent AuNPs, and black connecting lines denote DNA strands.

Prior to incubation with DNA, AuNP suspensions were concentrated as follows: 15 nm AuNPs: Approximately 60 mL of AuNP suspension was concentrated using 15 mL Amicon Ultra centrifugal filter units (100 kDa cutoff). The samples were centrifuged at 2000 rpm for 5 min at 20 °C. The retained particles were collected from the unit. 25 nm AuNPs: Approximately 60 mL of AuNP suspension was divided into four 15 mL Corning tubes and centrifuged at 4000 rpm for 1 h at 20 °C using a 5810-r Eppendorf centrifuge. The resulting pellets were resuspended in a minimal volume of the supernatant and combined. The final volume of concentrated particles of each size was ~100–200 μL, with an optical density (OD) of ~300–400 at 520 nm. To the concentrated AuNP suspensions, 5 mM BSPP was added, and the mixtures were incubated at ambient temperature for 1 h. Subsequently, KCl and HEPES-K (from 1 M stock solutions) were added to final concentrations of 15 mM and 5 mM, respectively, followed by vigorous mixing. Finally, dsDNA was added at a typical AuNP-to-DNA molar ratio of 1:1 for 15 nm and 1:2 for 25 nm NPs. For example, 15 nm AuNPs: 1 μM AuNPs (OD_520_ = 270) were mixed with 1 μM dsDNA. The corresponding OD_260_ values for 100 bp and 38 bp DNA were 0.33 and 0.12, respectively. 25 nm AuNPs: 0.4 μM AuNPs (OD_525_ = 300) were mixed with 0.8 μM dsDNA. The corresponding OD_260_ values for 100 bp and 38 bp DNA were 0.13 and 0.05, respectively. The incubation was carried out for approximately 16 h at RT. The resulting dumbbell structures were separated from excess AuNPs and from minor amounts of higher-order assemblies (comprising three or more nanoparticles) by electrophoresis in a 2% agarose gel. Approximately 15 µL of the incubation mixture was loaded into each lane, and electrophoresis was conducted for about one hour, as described in the Section 2. Figure 1 shows a gel image illustrating the separation of dumbbell structures composed of 15 nm AuNPs and 100 bp DNA. The colored gel band corresponding to the dumbbells, indicated in Figure 1 by the arrow above the AuNP band, was excised with a razor blade and transferred into a dialysis bag containing buffer solution for electroelution.

**Figure 2:**
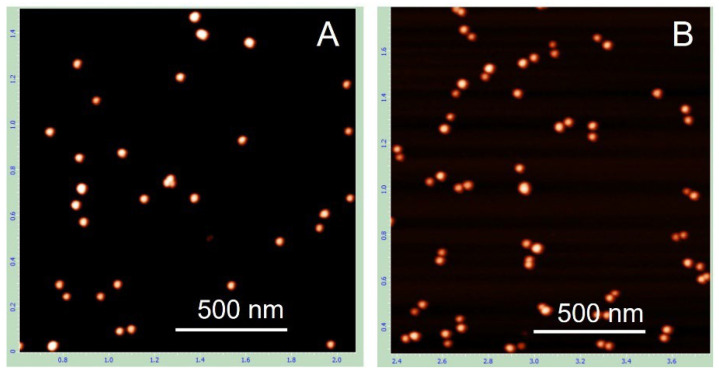
AFM images of AuNP–100 bp DNA dumbbells electroeluted from the gel under different conditions. The dumbbells were extracted from the gel region corresponding to their position (see Figure 1) into a dialysis bag containing either TAE buffer (**A**) or 1 mM citrate-K (pH 6.0) (**B**). The electroeluted conjugates were then concentrated by centrifugation, deposited onto PL-mica, and imaged by AFM as detailed in Section 2.

Notably, when the same TAE buffer used for electrophoresis was also employed for electroelution, the resulting preparation was characterized by a high percentage (68%) of single AuNPs (Table 1) as evident from the AFM image analysis (Figure 2). In contrast, using 1 mM potassium citrate buffer (pH 6.0) inside the dialysis bag and 5 mM potassium citrate buffer (pH 6.0) in the surrounding solution reduced the percentage of monomeric particles to 26%, with the majority of structures being nanoparticle-DNA dumbbells. The difference in the proportions of monomers and dimers in these preparations is evident from the images. In the preparation eluted in TAE buffer, most structures are individual, unconnected AuNPs (Figure 2A), whereas in the preparation eluted in 1 mM citrate, most structures are nanoparticle dumbbells (Figure 2B). Similar results, with slight variations in dimer and monomer content, were obtained in five independent experiments. The average dimer content in preparations eluted in citrate and TAE buffers was 74 ± 8% and 31 ± 5%, respectively.

This result highlights the importance of electroelution conditions for preserving the native state of the dimers. TAE buffer, though commonly used for elution of DNA from gels, appears to promote dissociation of the dimers into single particles, even under gentle electroelution conditions, namely, low temperature (with the electroelution bath immersed in ice water) and relatively low voltage (50 V). Substituting TAE buffer (40 mM Tris-acetate, pH ~8.5) with 1 mM citrate-K buffer (pH 6.0) significantly improved the yield of dimers composed of two AuNPs bridged by a DNA duplex (Table 1). The underlying mechanism of this effect is not yet clear. Maintaining low ionic strength during elution from the gel may be essential for preserving dumbbell integrity. Supporting this idea, increasing the citrate-K concentration from 1 mM to 10 mM led to a noticeable decrease in the dimer fraction. In addition, electroelution into 1 mM HEPES-K buffer (pH 7.5) produced a dimer fraction comparable to that obtained with 1 mM citrate-K. These observations further indicate that low ionic strength, rather than pH alone, is critical for preserving the structure of the dumbbells during elution. Regardless of the precise mechanism, these results clearly demonstrate that the use of a low-salt buffer during electroelution is essential for the preparation of native AuNP-DNA dumbbells.

The main goal of this study was the preparation of highly pure AuNP-DNA dumbbells composed of relatively large gold nanoparticles (15–25 nm in diameter) and short ds DNA (≤100 bp). This cannot be achieved using the common strategy where a gold nanoparticle is first conjugated with single-stranded DNA (ssDNA), and the resulting conjugate is then hybridized with a complementary particle-ssDNA conjugate. We have shown that conjugates of 15 nm AuNPs with 100-base ssDNA cannot be effectively separated from excess unbound nanoparticles using agarose gel electrophoresis (Figure S1). This is attributed to the negligible contribution of the DNA strand to the electrophoretic mobility of the comparatively large nanoparticle. In contrast, electrophoretic separation of conjugates of smaller AuNPs (5–10 nm) with longer DNA strands (≥100 bp) is primarily governed by the DNA. To overcome the challenges associated with separating DNA conjugates containing relatively large AuNPs, we adopted the following alternative strategy. A dsDNA molecule functionalized with thiol groups at both ends, obtained by annealing two complementary strands bearing 5′-terminal thiols, was incubated with an excess of AuNPs and then subjected to electrophoretic separation. The DNA-to-nanoparticle ratio in these experiments was kept low (≤1), to minimize the formation of multivalent conjugates (i.e., nanoparticles bound to multiple DNA molecules). Under these conditions, the predominant species in the mixture are unbound AuNPs, monovalent DNA-AuNP conjugates (one particle per DNA), and DNA-AuNP dimers (dumbbells). As shown in the gel electrophoresis image (Figure 1), the intense lower band corresponds to a mixture of unbound nanoparticles and monovalent conjugates, while the narrower band just above it corresponds to the dumbbells. Increasing the DNA-to-AuNP ratio results in the appearance of additional discrete bands, corresponding to higher-order assemblies visible as a ladder on an agarose gel (Figure S2). Incubation of the particles with the same DNA sequence lacking thiols under identical conditions did not result in dumbbell formation (Figure S6). This emphasizes that the particles are linked to the DNA specifically through the thiol groups at the ends of the double helix.

**Figure 3:**
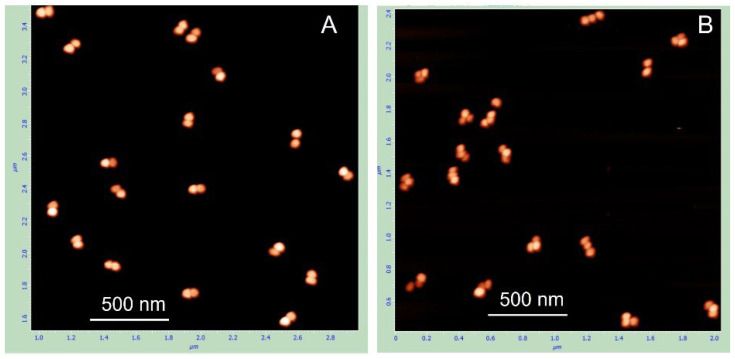
AFM images of AuNP–DNA dimers (**A**) and trimers (**B**). The conjugates were isolated from the gel regions corresponding to the dimers (**A**) and the trimer (**B**) (see Figure S2) and imaged by AFM following the protocol described in Section 2.

For any given band containing (n) AuNPs, the adjacent bands below and above contained (n – 1) and (n + 1) particles, respectively. AFM imaging of the structures electroeluted from the corresponding gel regions (Figure S2) revealed that the band located above the dumbbell band (Figure 3A) corresponds to a trimeric structure composed of three DNA-linked nanoparticles (Figure 3B). These results indicate that, in our system, the electrophoretic mobility of the conjugates is primarily determined by the number of AuNPs in the complex rather than the length of the DNA. This is further supported by the nearly identical electrophoretic mobility of dumbbells composed of 15 nm AuNPs and either 100 bp or 38 bp dsDNA (Figure S3). As shown in the gel image (Figure S3), the bands corresponding to these dumbbells migrate similarly, regardless of DNA length.

**Figure 4:**
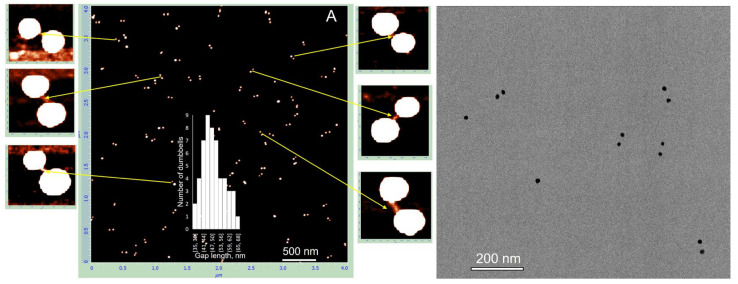
AFM and TEM analysis of 15 nm AuNP–100 bp DNA dumbbells. The conjugates were isolated from the gel as described in the Section 2. AFM imaging (**A**) and TEM imaging (**right panel**) were performed as detailed in the Materials and Methods. The side panels in (**A**) show magnified views of the structures indicated by yellow arrows.

We prepared and characterized gold nanoparticle dumbbell structures composed of either 15 nm or 25 nm AuNPs linked by dsDNA of 100 or 38 bp, using the above experimental strategy (see Section 2). All dumbbells migrated as distinct bands on agarose gels and appeared as discrete dimeric structures in both AFM and high-resolution TEM images. Representative AFM and TEM images of the dumbbells composed of 15 nm AuNPs and 100 bp DNA are shown in Figure 4A,B, respectively. The calculated average center-to-center distance between nanoparticles in the dimer is 50 ± 5 nm (inset in Figure 4A). This agrees well with the expected value based on a 100 bp dsDNA length (34 nm) plus the nanoparticle diameter, yielding ~49 nm, which closely matches the measured distance. The calculated interparticle distance of ~30 nm agrees well with the TEM measurements (Figure 4B); the edge-to-edge distance between nanoparticles, corresponding to the length of the DNA bridge, is also about 30 nm. The advantage of AFM over TEM is that it allows visualization of both the particles and the DNA forming the dimer. In the magnified AFM images of individual dumbbells (panels adjacent to Figure 4A), a DNA fiber connecting the two particles is clearly visible.

**Figure 5:**
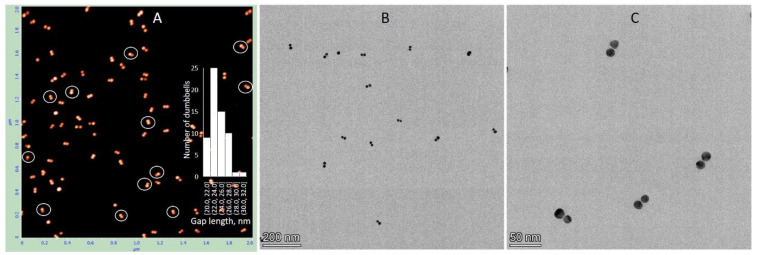
AFM and TEM analysis of 15 nm AuNP–38 bp DNA dumbbells. The conjugates were isolated from the gel as described in Section 2. AFM imaging (**A**) and TEM imaging (**B**,**C**) were performed as detailed in Section 2. The inset in (**A**) shows statistical analysis of the dimer gap lengths.

Similar characterization was performed for dumbbells composed of 15 nm AuNPs with 38 bp DNA, 25 nm AuNPs with 100 bp DNA, and 25 nm AuNPs with 38 bp DNA. The electrophoretic separation patterns of all these structures were similar (Figure S4). Dumbbell structures of all the above types were electroeluted from gel regions as described in Section 2 and characterized by AFM and TEM. The results for the dumbbell composed of 15 nm AuNPs and 38 bp DNA showed that the interparticle distance measured by AFM closely matched the expected length of the 38 bp DNA (~13 nm) connecting the particles. The average center-to-center distance between the nanoparticles was 24.5 ± 5 nm (inset in Figure 5A), which corresponds to a DNA length of approximately 9.5 nm after subtracting the 15 nm nanoparticle diameter from the measured value. However, the interparticle distance measured by TEM was significantly shorter, approximately 1 nm (Figure 5B,C). This suggests that the dumbbells collapsed on the TEM grid during deposition and/or drying, causing the DNA connecting the particles to lose its rigid structure and the nanoparticles to come into almost direct contact. This process also occurs on mica; however, the proportion of collapsed dumbbells remains relatively low, not exceeding 15%. These collapsed dimers are marked with white circles in the AFM image (Figure 5A). Pretreatment of the mica surface with PolyLysine (PL) results in the formation of a positively charged polymer layer, enhancing the adsorption of negatively charged biomolecules through electrostatic attraction. Electrostatic binding of the structures, followed by rapid washing with cold DDW and drying, thus appears to be a less damaging procedure compared to adsorption onto a carbon grid during drying. Dumbbells composed of 25 nm AuNPs and 100 bp DNA exhibit similar behavior.

**Figure 6:**
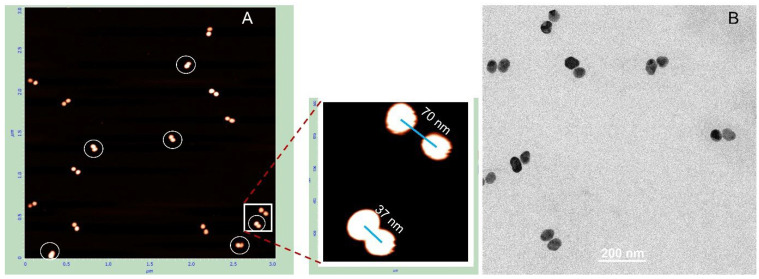
AFM and TEM analysis of 25 nm AuNP–100 bp DNA dumbbells. The conjugates were isolated from the gel as described in the Section 2. AFM imaging (**A**) and TEM imaging (**B**) were performed as detailed in Section 2. The side panel in (**A**) presents magnified views of the structures highlighted by the white rectangle.

**Figure 7:**
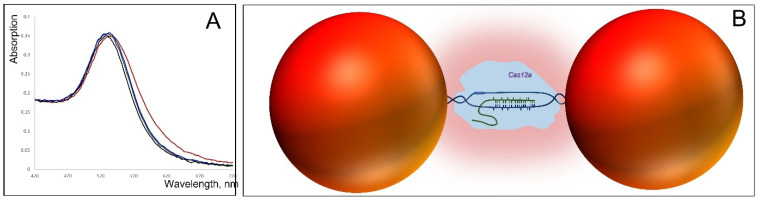
Interaction of CRISPR-Cas12 with 25 nm AuNP–38 bp DNA dumbbells. (**A**) Absorption spectra of the dumbbell alone (black), mixed with 25 nM Cas12–guide RNA (red), 25 nM Cas12–guide RNA conjugated with complementary dsDNA (blue), and 100 nM BSA (green) in 20 mM HEPES-K (pH 7.5) containing 20 mM KCl. Spectra were recorded 5 min after mixing the dumbbell with the proteins (Cas12 or BSA). The preparation of the dumbbell is described in Section 2; the preparation of CRISPR-Cas12 and its DNA conjugate is detailed in Section 2 (**B**) Schematic illustration of Cas12 interaction with the dumbbell. The enzyme (light blue), carrying guide RNA (green curve), searches for a complementary sequence within the dumbbell’s DNA. Upon binding, local DNA melting (blue curves) occurs, allowing the unwounded DNA strand to partially hybridize with the guide RNA. This leads to the formation of a protein–DNA–RNA complex that bridges the gap between the particles (red spheres), creating an environment that enhances interparticle interactions (depicted as a pink cloud).

Deposition on PL-treated mica results in approximately 40% collapsed dimers, where the nanoparticles are almost directly connected. These dimers are indicated by white circles in Figure 6A. As shown in the magnified image adjacent to panel A, the distance between particles in native (non-collapsed) dimers is considerably longer than in collapsed ones and corresponds to the length of 100 bp DNA. The edge-to-edge distance between particles in collapsed dimers (bottom-left corner of the magnified image in Figure 6) is about 12 nm. We were unable to identify any dumbbells on the TEM grid exhibiting an expected gap of about 30 nm between particles. In all dimers observed by TEM, the particles were almost directly connected with an edge-to-edge separation of less than 2 nm. This further emphasizes the importance of the deposition procedure for preserving the native DNA structure in these conjugates. To exclude the possibility that a fraction of the dumbbells collapsed already in solution, we examined their photophysical properties. We measured the absorption spectrum of 25 nm AuNP–38 bp dumbbells (composed of 25 nm AuNPs and 38 bp DNA). The interparticle gap of approximately 12 nm is consistent with the length of 38 bp dsDNA. The interaction between the particles results in only a negligible shift in the plasmonic wavelength maximum compared with unlinked AuNPs (compare black and blue curves in Figure 7A). This observation is consistent with theoretical calculations showing no significant plasmonic interaction between even larger particles (50 nm) when separated by more than 5 nm in a dimer [8]. In contrast, we previously found that closely spaced (1–2 nm) 15 nm AuNP dimers exhibited an additional absorption peak at ~570 nm alongside the main plasmon resonance at 520 nm [24]. Similarly, 25 nm AuNP dimers with very short interparticle gaps, prepared as described in Materials and Methods, display a pronounced secondary absorption band centered near 600 nm (Figure S5), indicative of strong plasmonic coupling. Notably, this secondary peak is absent in DNA–AuNP dumbbells, suggesting that no collapsed dimers (with particles nearly in direct contact, as observed in TEM images; Figure 5 and Figure 6) are present in aqueous solution.

The plasmonic interaction is influenced by the properties of the interparticle medium. We have previously shown that linking metal nanoparticles with G4-DNA molecules promotes plasmonic coupling [7], likely due to the higher electrical conductivity of G4-DNA compared to dsDNA. Thus, the ability of DNA-AuNP dumbbells to conduct either current or electromagnetic signals depends strongly on the properties of the medium separating the particles. This makes it possible to observe enzyme binding to the dumbbell at the single-molecule level. Moreover, binding of enzymes to the DNA bridge could be exploited to track conformational changes during catalytic turnover, either by monitoring conductivity through the structure or by detecting changes in capacitance [12].

To explore the effect of DNA-dependent enzyme binding on dumbbell properties, we chose the CRISPR–Cas12 enzyme [25,26], not only because of its popularity in biomedical research, but mainly because, in its search mode, Cas12 interacts with dsDNA through hybridization of its guide RNA. This RNA-containing enzyme is ~7–8 nm in size, meaning that its binding would fill the gap between AuNPs in a dumbbell composed of 25 nm particles bridged by 38 bp DNA almost completely (Figure 7B). Indeed, the addition of Cas12 produced a noticeable red shift of the absorption maximum, accompanied by changes in spectral shape (Figure 7A, black and red curves). By contrast, no spectral changes were observed when Cas12 was pre-bound to its target DNA and thus unable to interact with other DNA molecules. Importantly, control experiments confirmed that the observed shift was not due to nonspecific protein adsorption on the particle’s surface, as no spectral effect was detected with BSA (Figure 7A, black and green curves). These results demonstrate that specific binding of Cas12 to the DNA bridge in the dumbbell alters plasmonic coupling between the AuNPs. This result paves the way for the development of a sensing unit in which conformational changes of an enzyme bound to the DNA bridge during catalysis modulate the dumbbell’s electrical (conductivity, impedance, capacitance) and optical properties, thereby enabling real-time monitoring of the enzyme’s catalytic cycle. All dumbbells examined in this study demonstrated high stability in solution. They remained intact without dissociating into single particles even after one month of storage in double-distilled water (DDW) at 4 °C and maintained stability for at least one week at 25 °C in DDW. The same percentage of dimers was estimated by AFM imaging as that measured in Figure 4 for freshly prepared dumbbells. This robust stability in pure water makes these structures particularly suitable for applications in molecular electronics, where the presence of salts could interfere with electrical behavior by introducing ionic currents. The main challenge in electrical measurements of the dumbbells is maintaining the integrity of the DNA connecting the particles. This is particularly challenging because electrode measurements typically require deposition and subsequent drying of the structures on the electrode surface, a process that can lead to DNA denaturation. To minimize such damage, measurements should ideally be carried out in solution or on electrodes modified with a positively charged organic polymer, allowing gentle electrostatic binding to a soft layer, similar to the PL-coated mica surfaces used in AFM measurements. In any case, the DNA structure is more likely to remain intact in dumbbells composed of smaller nanoparticles and longer DNA linkers. However, it should be noted that the nucleic acid structure can be altered during deposition and subsequent drying of the dumbbells. In aqueous environments, the complementary strands of dsDNA (both carrying negative charges) experience electrostatic repulsion that can overcome hydrogen bonding between base pairs, leading to strand separation and disruption of the double helix. Rinsing the electrodes bearing the deposited dumbbells with water, a step necessary to remove salts and buffer components, may cause significant damage to the DNA. While rapid rinsing with cold water might reduce this effect, the preservation of the native DNA conformation in the dumbbell remains questionable. This challenge can be addressed by employing DNA-based structures that are inherently more stable than the canonical double helix, such as DNA triplexes, G-quadruplexes (G4-DNA) or DNA–PNA hybrids. The latter are particularly stable in pure water because the peptide nucleic acid (PNA) strand is uncharged, so no electrostatic repulsion between the two strands occurs even in deionized water. G4-DNA can also resist the treatment, since this structure is significantly more stable than dsDNA [27,28]; continuous G-strands resist denaturation even upon boiling in pure water [29,30]. Furthermore, guanine has the lowest redox potential among the nucleobases, which can facilitate electron transfer through the DNA. We have previously shown that G4-DNA and G8-DNA [31,32] exhibit high electrical polarizability [29,30]. Such dumbbells, in which two gold nanoparticles are bridged by a stable, rigid, and conductive DNA molecule, hold significant potential for advancing biosensor and bioelectronic device technologies.

## Figures and Tables

**Table 1:** Effect of elution buffer composition on dimer yield.

Preparation Name	Number of Dimers	Number of Monomers	% Dimers *
PREP-1 (1 mM Cit)	217	80	73
PREP-2 (TAE)	108	230	32

* More than 200 structures from AFM images (see Figure 2) were analyzed.

## Data Availability

The original contributions presented in this study are included in the article/Supplementary Material. Further inquiries can be directed to the corresponding author.

## Supplementary Materials

The following supporting information can be downloaded at: https://www.mdpi.com/article/10.3390/nano15201583/s1, Figure S1. Electrophoretic separation of 15 nm AuNP–100 b ssDNA conjugates; Figure S2. Electrophoretic separation of 38 bp DNA- 25nm AuNP conjugates prepared at different particle-to-DNA ratios; Figure S3. Electrophoretic separation of 15 nm AuNP dumbbells with DNA of different lengths; Figure S4. Electrophoretic separation of various AuNP–DNA dumbbells; Figure S5. Electrophoresis and absorption spectroscopy of nanoparticle structures; Figure S6. Formation of AuNP dumbbells requires thiol groups at the DNA ends.
