# Supplementary material for: DNA–Gold Nanoparticle Dumbbells: Synthesis and Nanoscale Characterization
Source: Nanomaterials (Basel). 2025 Oct 17;15(20):1583. doi: 10.3390/nano15201583 (PMC12566764; doi:10.3390/nano15201583)
Supplement: Supplementary file 1 [file nanomaterials-15-01583-s001.zip › nanomaterials-3878282-supplementary.pdf]

Supporting Information

DNA–Gold Nanoparticle Dumbbells: Synthesis and Nanoscale Characterization

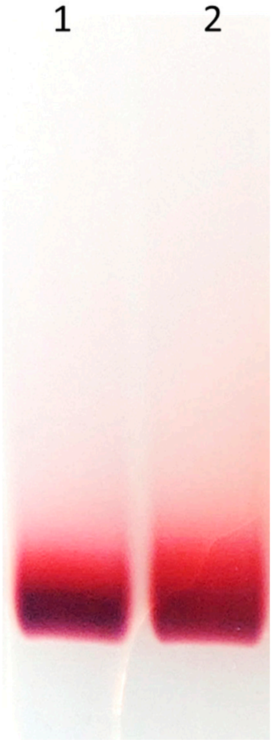

Figure S1. Electrophoretic separation of 15 nm AuNP–100 b ssDNA conjugates. Lane 1: 0.25 μM 15 nm AuNPs. Lane 2: 0.3 μM 15 nm AuNPs incubated with 0.1 μM 100 b ssDNA ("100-1"; see Materials and Methods for sequence) for 16 h at RT in 5 mM HEPES–K (pH 7.5), 15 mM KCl. Electrophoresis was carried out in 2% agarose gel (7 × 7 cm^2) in TAE buffer at 100 V for 1 h in an ice bath, as described in Materials and Methods.

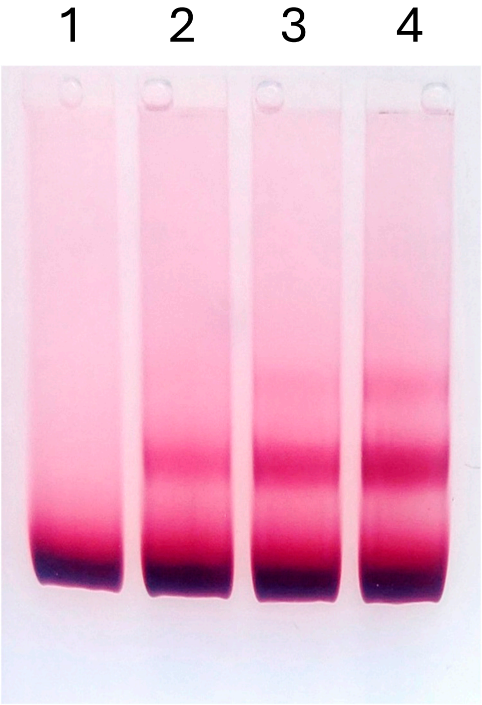

Figure S2. Electrophoretic separation of 38 bp DNA-25 nm AuNP conjugates prepared at different particle-to-DNA ratios. Lane 1: 0.05 μM 25 nm AuNPs. Lanes 2–4: 0.05 μM 25 nm AuNPs incubated with 0.02, 0.05, and 0.1 μM 38 bp DNA, respectively, for 16 h RT in 5 mM HEPES–K (pH 7.5), 15 mM KCl. Electrophoresis was carried out in 2% agarose gel (7 × 7 cm^2) in TAE buffer at 100 V for 1 h in an ice bath, as described in Materials and Methods.

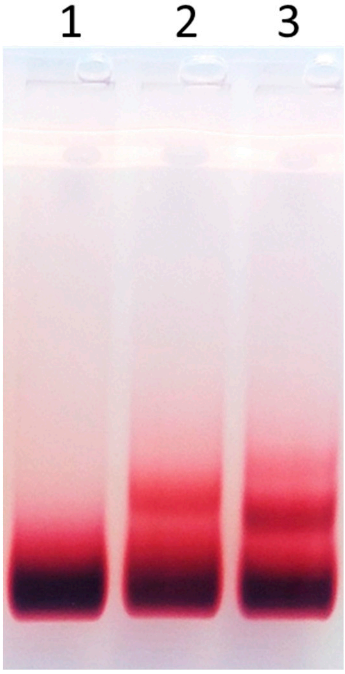

Figure S3. Electrophoretic separation of 15 nm AuNP dumbbells with DNA of different lengths. Lane 1: 15 nm AuNPs. Lane 2: AuNP–100 bp DNA dumbbells. Lane 3: AuNP–38 bp DNA dumbbells. Conjugate preparation and electrophoresis were carried out as described in Materials and Methods.

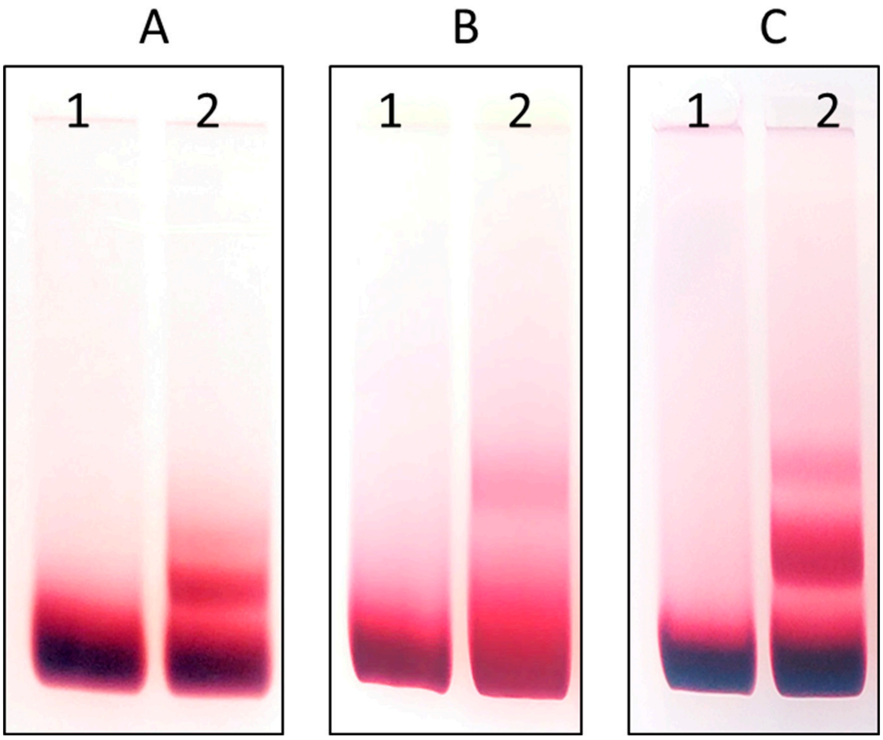

Figure S4. Electrophoretic separation of various AuNP–DNA dumbbells. (A) 15 nm AuNPs with 38 bp DNA; (B) 25 nm AuNPs with 100 bp DNA; (C) 25 nm AuNPs with 38 bp DNA. Conjugate preparation and electrophoresis were performed as described in Materials and Methods.

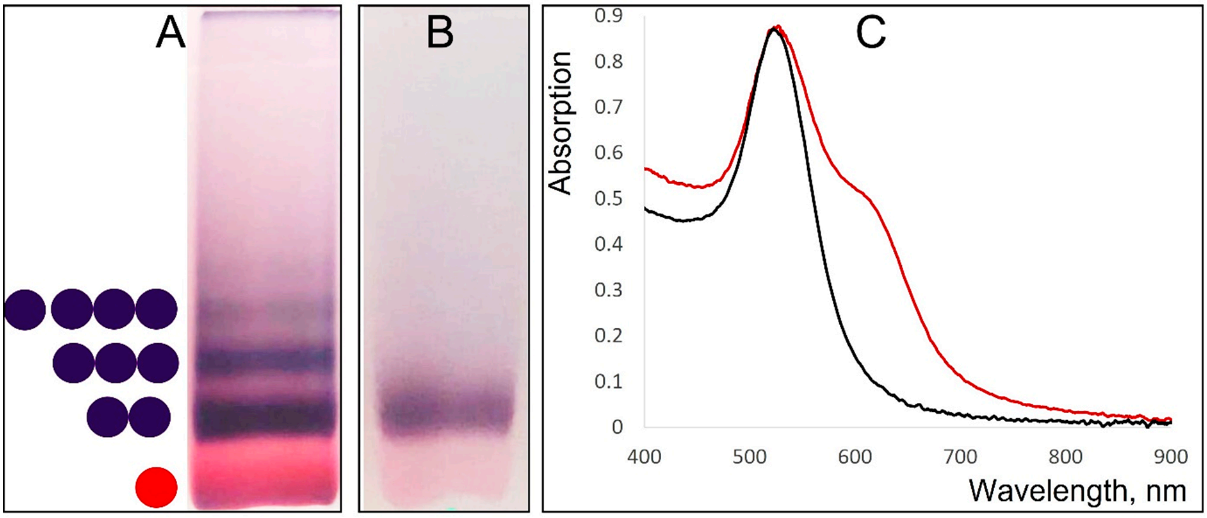

Figure S5. Electrophoresis and absorption spectroscopy of nanoparticle structures. (A) Closely spaced 25 nm AuNPs structures were prepared and electrophoresed as described in Materials and Methods. A schematic representation of the structures corresponding to the gel bands is shown to the left of the lane. (B) The dimer fraction was electroeluted from the gel, concentrated by centrifugation, and reloaded onto the gel. (C) Absorption spectra of the monomeric (red curve) and dimeric (magenta curve) fractions, both electroeluted from their respective gel regions.

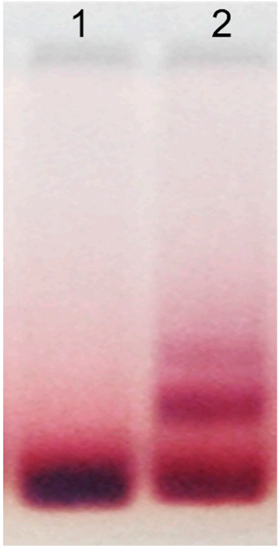

Figure S6. Formation of AuNP dumbbells requires thiol groups at the DNA ends. 15 nm AuNPs were incubated with 100 bp DNA either lacking thiols (Lane 1) or containing thiols at both ends of the double helix (Lane 2) as described in Materials and Methods. Electrophoresis was also carried out as described in Materials and Methods.
